# Eutectic Fatty Acids Phase Change Materials Improved with Expanded Graphite

**DOI:** 10.3390/ma15196856

**Published:** 2022-10-02

**Authors:** Zanshe Wang, Guoqiang Huang, Zhaoying Jia, Qi Gao, Yanping Li, Zhaolin Gu

**Affiliations:** School of Human Settlement and Civil Engineering, Xi’an Jiaotong University, Xi’an 710049, China

**Keywords:** phase change materials, eutectic fatty acids, expanded graphite, ultra-low-grade energy, thermal energy storage

## Abstract

Low- and ultra-low-grade thermal energy have significant recycling value for energy saving and carbon footprint reduction. Efficient thermal energy storage technology based on phase change materials (PCMs) will help improve heat recovery. This study aimed to develop a composite eutectic fatty acid of lauric acid (LA) and stearic acid (SA) binary system with expanded graphite (EG). The experimental measured eutectic temperature was 31.2 °C with an LA-to-SA mass ratio of 7:3. Afterwards, 1~15 wt.% EG was composited to the eutectic acid, and the thermophysical properties of the composite PCMs were measured by differential scanning calorimetry (DSC) and transient plane source (TPS) methods. The results demonstrated that the phase transition temperature and latent heat of the composite PCMs were stable when the content of EG was more than 5%, and the thermal conductivity and thermal diffusion coefficient of the composite PCMs (10–15 wt.%) increased by 2.4–2.6 and 3.2–3.7 times compared with the pure eutectic acid, respectively. On this basis, a finned-coil-type reservoir was prepared, and an experimental study of heat storage and heat release performance was carried out. The results showed that the heat storage and heat release effects of the heat reservoir were the best when the EG ratio was 10 wt.%. The heat storage time was reduced by 20.4%, 8.1%, and 6.2% compared with the other three EG ratios, respectively; meanwhile, the heat release time was reduced by 19.3%, 6.7%, and 5.3%, respectively.

## 1. Introduction

**Figure 1:**
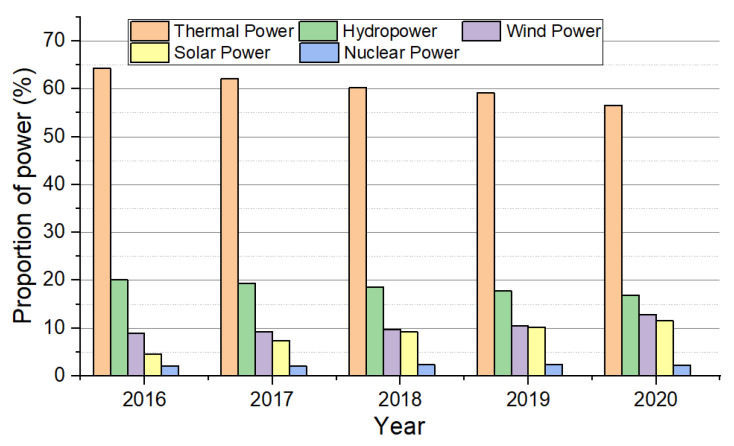
The proportion of China’s energy structure in the past five years.

The problems of energy shortage and global warming have become worldwide research hotspots. It has become a consensus to increase the proportion of renewable energy and clean energy, improve energy utilization efficiency, reduce carbon emissions, and achieve the sustainable development of energy and the environment. In recent years, although new powers such as solar, wind, geothermal, hydraulic, biomass, and nuclear energy have rapidly developed, they still account for a small proportion of the total energy in China [1], as shown in Figure 1. Therefore, vigorously developing renewable energy, optimizing the energy structure, improving energy utilization efficiency, and entirely using waste heat resources have become necessary technical means for energy conservation and emission reduction.

Thermal energy storage (TES) technology can improve energy utilization efficiency and effectively alleviate the mismatch between the energy supply and demand in time, space, and intensity [2]. Generally, TES can be divided into four categories: sensible heat storage, latent heat storage, thermochemical heat storage, and adsorption heat storage [3]. Among them, latent heat storage is the most widely used due to its advantages of large heat storage capacity and small heat storage volume [4]. The key to latent heat storage is the selection and preparation of the phase change materials (PCMs). Inorganic and organic PCMs with different phase change temperatures have been extensively studied [5]. The shortcomings of supercooling and phase separation of inorganic PCMs are perfected by adding thickeners [6], and the weakness of the low thermal conductivity of organic PCMs is improved by adding microparticles with high thermal conductivity, such as graphite [7,8,9,10,11], silicon dioxide [12,13,14,15], expanded graphite [16,17,18,19,20,21], etc.

Moreover, to modulate the PCMs with specific phase change temperatures, the composite preparation of different phase change materials is often adopted, and this physical mixing preparation method enables a broader application for the PCMs. Zalba [2] listed over 150 materials used in research as PCMs, which has focused on the materials, heat transfer, and applications. Pielichowska [22] presented the improvement in thermal conductivity, encapsulation methods, and shape stabilization procedures; Yang [4] discussed PCM property characterization and the need for materials design; and Gracia [23] introduced the materials used for building thermal energy storage. Demirbas [24] presented the detailed thermophysical property parameters of the main PCMs: paraffin, inorganic compounds, inorganic eutectics, and organic compounds. Ammar [25] proposed the low-grade heat sources in major industrial processes in the U.K., which showed that wastewater temperature was about 45–50 °C.

Furthermore, the thermal energy grades are usually classified into three levels based on the temperature of heat sources [26,27]: high-grade (higher than 650 °C), medium-grade (230–650 °C), and low-grade (lower than 230 °C). Generally, the higher the temperature of the heat source, the easier the recovery and the higher the thermal efficiency.

Although heat sources less than 230 °C are uniformly classified as low-grade heat sources, the grade of energy can be further reclassified. When the heat source temperature is higher than 120 °C, the lithium bromide absorption refrigeration and heat pump system [28,29] and the organic Rankine cycle [30,31,32] can be used to achieve the efficient recovery and utilization of heat energy. When the heat source temperature is higher than 80 °C, the waste heat boiler [33] and the heat exchanger [34] were used to improve the grade and utilization efficiency of the heat source. When the heat source temperature is around 50 °C, the heat pump technology [35] and energy system optimization method [36] have been widely used. Notably, low-temperature heat sources are suitable for building heating [37,38,39].

Therefore, it can be seen that the lower the temperature of the heat source, the lower the heat recovery efficiency [27]; conversely, the greater the number of heat sources, the greater the recovery potential [40,41]. Low-temperature heat sources with a temperature below 50 °C are widely distributed, such as industrial waste heat, solar hot water, waste heat from waste gas and wastewater, and circulating cooling water. Low-temperature heat sources are extensive and have good recovery value. As a result, low-temperature heat sources can be called ultra-low grade thermal energy. Due to the low temperature, thermal energy storage can be carried out utilizing phase PCM heat storage as a low-grade energy pool to achieve total energy savings for the system. Low- and ultra-low-grade thermal energy are especially suitable as the low-end energy source of heat pumps. It was verified that their use in industry and buildings will reduce energy consumption and improve the economy [42].

Consequently, because the temperature of the ultra-low-grade thermal energy is low, the PCMs used for low-temperature heat sources should have two characteristics: significant melting and solidification enthalpy, and good thermal conductivity and heat exchange performance. The fatty acid PCMs belong to the nonparaffinic organic compounds, which perform better than other PCMs. Some advantages of fatty acid PCMs include their comelting and cocrystallization characteristics, high melting enthalpy, incombustibility, small volume expansion rate in the process of solid–liquid phase transition, low cost and obtainability, and good thermal and chemical stability after cold and hot cycle tests [43,44,45,46]. Among the fatty acids, lauric acid (LA), stearic acid (SA), myristic acid (MA), and palmitic acid (PA) are the most commonly used PCMs that can be compounded with each other to produce novel PCMs with different phase change temperatures [47]. Sarı [48] tested the thermal properties of three eutectic mixtures: LA–MA (34.2 °C melting point in 66.0:34.0 wt.%), LA–PA (35.2 °C melting point in 69.0:31.0 wt.%), and MA–SA (44.1 °C melting point in 64.0:36.0 wt.%) by using a differential scanning calorimeter (DSC). Liu [49] proposed a ternary eutectic mixture and expanded graphite composite PCM of LA-MA-SA/EG (12:1) with a 29.05 °C melting point and 29.38 °C freezing point.

As mentioned above, low-temperature heat sources below 50 °C determine the lower phase change temperature of PCMs. Therefore, it is imperative to prepare specific PCMs suitable for low temperatures and test their performance in practical applications. In this study, we chose LA, SA, and expanded graphite (EG) to form binary eutectic PCMs. A finned-coil-type reservoir was prepared by filling the composite PCMs. The experimental tests were carried out to study the performance of composite PCMs in ultra-low grade thermal energy storage.

## 2. Materials and Methods

### 2.1. Materials

The lauric acid (analytical reagent grade of 98% purity) was obtained from Tianjin Hebei Haijing Fine Chemical Factory, Tianjin, China, and the stearic acid (analytical reagent grade of 97% purity) was obtained from Chengdu Kelong Chemical Reagent Factory, China. The expanded graphite powder (325 mesh, thermal conductivity: 90 W·m^−1^·k^−1^) was obtained from Qingdao Tianshengda graphite Co., Ltd., Qingdao, China. All the raw materials were used as received from the suppliers without any further purification. Table 1 shows the basic parameters of the lauric acid (LA) and stearic acid (SA) provided by the suppliers.

### 2.2. Preparation and Process Methods

Due to the excellent miscibility of fatty acids, the lauric and stearic acids can be mixed to obtain the eutectic system of binary fatty acids, and the melting point of materials can be reduced to get the needed PCMs. More importantly, eutectic mixtures are the most stable because intermolecular forces combine the components.

**Figure 2:**
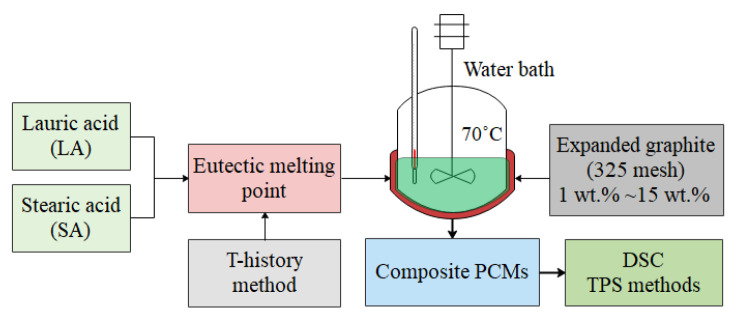
The preparation process of composite PCMs.

**Figure 3:**
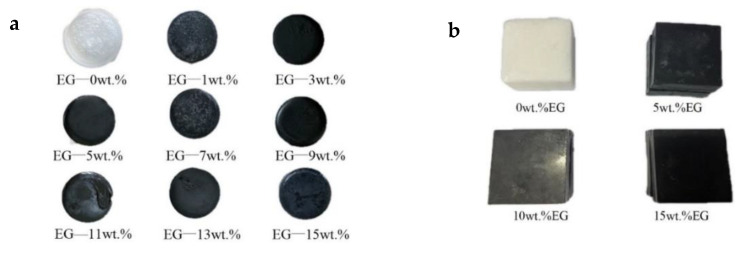
(**a**) DSC test samples; (**b**) Thermal conductivity test samples.

The preparation process of composite PCMs is shown in Figure 2. Firstly, the binary eutectic point of LA and SA was tested by the step cooling curve experiment method. The LA and SA were thoroughly mixed in different proportions and heated to complete melting, and then the cooling experiment was carried out to obtain the step cooling curve under different mixing ratios and determine the binary eutectic temperature point. Secondly, different proportions of expanded graphite (325 mesh) were gradually added to the melted binary eutectic mixed acid at 70 °C; at the same time, the agitator was stirred for about 1 h at 15–30 r/min until complete mixing; finally, the entire mixture was cooled in the water bath at 20.0 °C to form the composite PCMs. The basic thermophysical parameters were measured by differential scanning calorimetry (DSC) and transient plane source (TPS) methods. As a result, 9 DSC and 4 thermal conductivity test samples were prepared with different EG ratios, as shown in Figure 3.

### 2.3. Differential Scanning Calorimetry (DSC)

A differential scanning calorimeter Discovery DSC250 (TA Instruments, NC, DE, USA, accuracy: ±0.05 °C) was used to test the melting point, freezing point, phase change enthalpy of PCMs, and other basic physical parameters. The test samples were weighed with a Mettler Toledo MS105DU electronic balance (Switzerland, accuracy: ±0.01 mg). The test samples were collocated in a pan, and the heating rate was 1 °C/min.

### 2.4. Transient Plane Source (TPS) Methods

A Hot Disk TPS-2500 S thermal constant analyzer (Sweden, accuracy: ±3%) was used. The transient plane source test method was implemented using a sandwich structure. The test sample size was 5.0 × 5.0 × 2.5 cm, as shown in Figure 3b.

### 2.5. Low-Temperature Thermal Energy Storage Experiment

#### 2.5.1. Finned-Coil-Type Heat Reservoir

In addition to the properties of PCMs, the structural form of the heat reservoir is also key to the phase change heat storage process, especially for low- and ultra-low-grade heat sources. Generally, the PCMs nearest to the heat or cold wall are the first to undergo a phase change, then heat is gradually transferred to the unchanged part by heat conduction and convection. As a result, the process of heat storage and heat release is significantly weakened, and the efficiency is reduced when only relying on the heat conduction of the phase change heat storage material to complete the heat storage and release.

**Figure 4:**
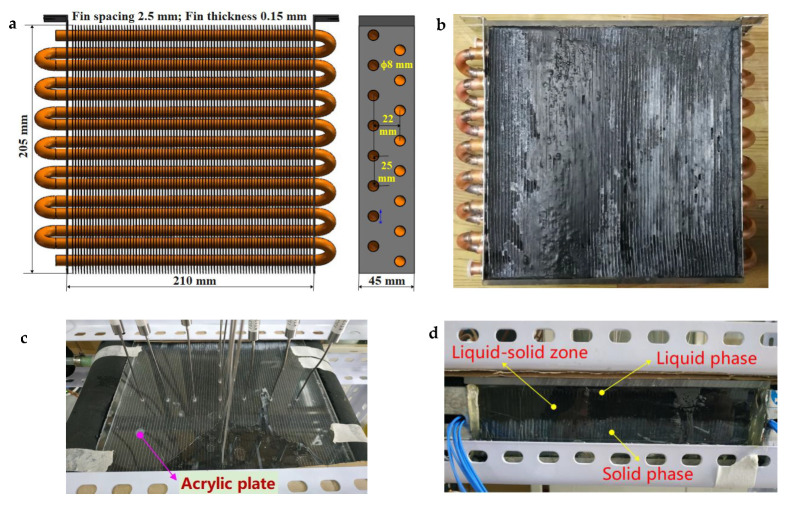
(**a**) Finned coil-type structure and parameters; (**b**) Filling with PCMs; (**c**) Temperature probe locations; (**d**) Observation surface.

Therefore, to test the rapid heat storage and release of low- and ultra-low-grade heat sources, a finned coil-type reservoir was prepared in this study. The material of the tube was brass (thermal conductivity: 108.9 W·m^−1^·k^−1^), and the fabric of the fin was 1060 aluminum (thermal conductivity: 234 W·m^−1^·k^−1^). The composite PCMs were filled in to study the heat storage and heat release performance. The detailed structure, parameters, and testing device are shown in Figure 4. The finned-coil-type reservoir with a 205 mm height and 210 mm width was wrapped and sealed with an acrylic plate, and then wrapped with insulation foam. Thirteen temperature sensors were arranged on the upward acrylic panel and inserted into the PCMs, the acrylic panel on one side was used to observe the melting and solidification state of PCM.

#### 2.5.2. Testing System

**Figure 5:**
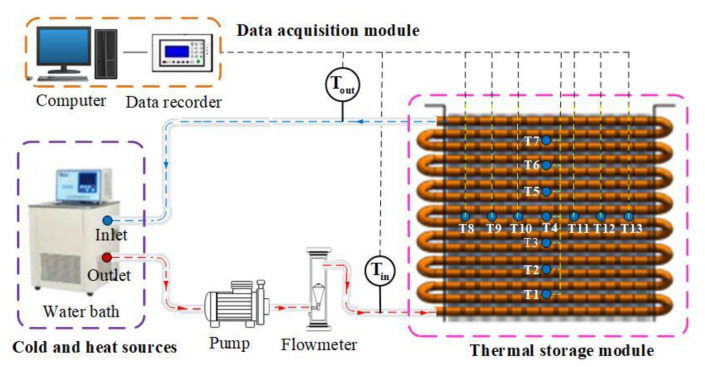
Schematic diagram of the experimental apparatus.

Figure 5 shows the schematic diagram of the experimental apparatus. A thermostatic water bath (FDLDHX-2050, Nanjing, China, accuracy: ±0.1 °C) provided the stable heat resources for the heat reservoir, and a rotameter (LZB-25F, Changzhou, China, 250–2500 L/h; accuracy: ±1.5%) ensured the stability of the flow. A total of 13 thermocouple sensors (Pt100, Shanghai, China, accuracy: ±0.2 °C) were uniformly inserted into the PCMs in a cross shape; another 2 thermocouple sensors measured the inlet and outlet water temperature. All thermocouple sensors were connected to the data recorder and measured every second.

Four identical finned coil-type heat exchangers were filled with the composite PCMs of 0 wt. %, 5 wt. %, 10 wt. %, and 15 wt. % for the experimental testing. The net weight of PCMs in 4 heat reservoirs is shown in Table 2. The values of the same filling amount were compared when analyzing the test data of the four heat reservoirs.

## 3. Results and Discussion

### 3.1. The Eutectic Point of LA and SA

**Figure 6:**
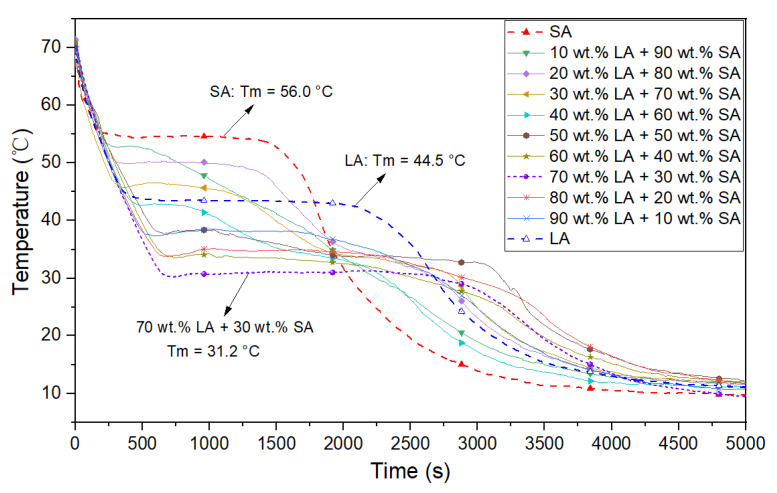
Experimental tests of the eutectic melting point of LA and SA.

Lauric and stearic acids with different mass ratios were prepared and evenly mixed, then put into a constant-temperature water bath at 70 °C. After being wholly melted and stable, they were put into a consistent-temperature environment at 10 °C for natural cooling, and the step cooling curve of PCMs was obtained, as shown in Figure 6. The eutectic melting point was 31.2 °C when the mass proportion of LA was 70 wt.% and that of SA was 30 wt.%. Moreover, the phase transition platform was stable and lasted longer. Therefore, this mixed acid was adopted as the basic PCM to composite with expanded graphite. As a result, the test samples shown in Figure 3 were prepared based on this mixed acid ratio.

### 3.2. DSC Results

**Figure 7:**
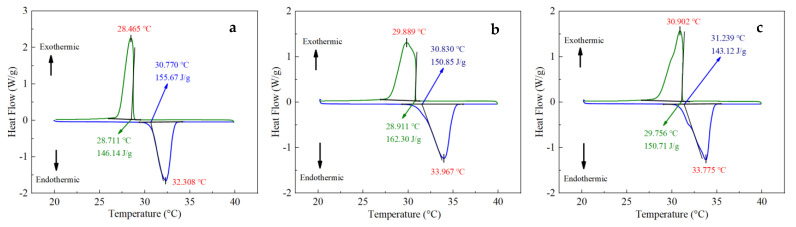

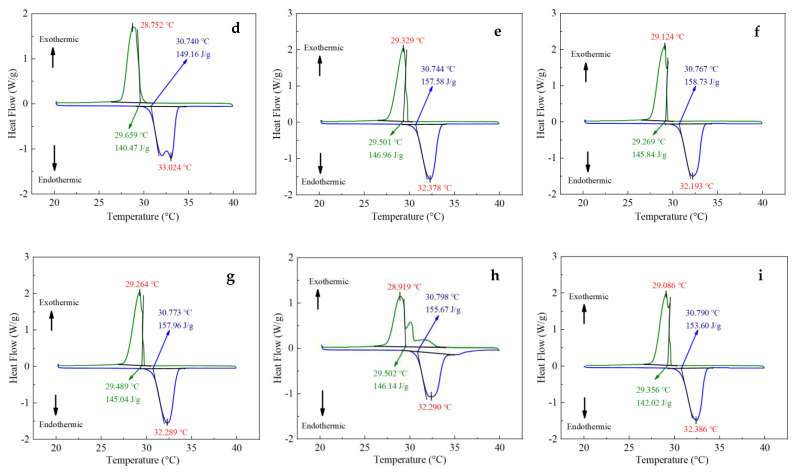
DSC curves of the composite PCMs: (**a**) 0 wt.% EG; (**b**) 1 wt.% EG; (**c**) 3 wt.% EG; (**d**) 5 wt.% EG; (**e**) 7 wt.% EG; (**f**) 9 wt.% EG; (**g**) 11 wt.% EG; (**h**) 13 wt.% EG; (**i**) 15 wt.% EG.

**Figure 8:**
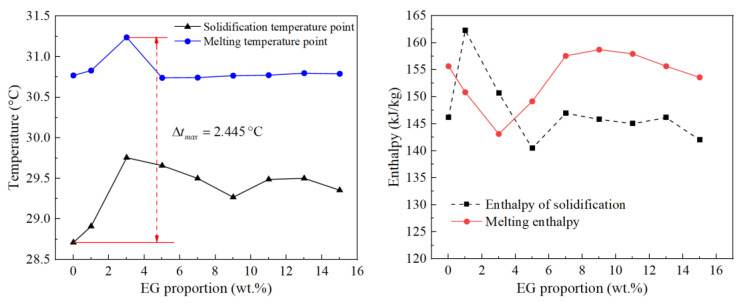
(**a**) Phase transition temperature; (**b**) Phase transition enthalpy.

Because the melting and freezing points of organic PCMs are different, both the endothermic and exothermic processes were carried out. The samples were heated from 20 to 40 °C and then cooled from 40 to 20 °C. Both the heating and cooling rates were 1 °C/min to ensure PCMs underwent the two processes of melting and solidification. Based on the recorded data, nine groups of DSC curves were obtained, including the starting temperatures, peak temperatures, and enthalpy of melting and solidification, as Figure 7 shows. According to the statistics of the DSC test data, the phase transition melting point and freezing point of PCMs are shown in Figure 8a, and the melting and freezing enthalpies of the PCMs are shown in Figure 8b.

It can be seen that with the increase in the proportion of expanded graphite, the melting point of PCMs was mostly stable at about 30 °C, while the solidification point fluctuated within the range of 28.7 to 31.2 °C, and the maximum temperature difference between the melting and the solidification point was 2.445 °C. The DSC test data were consistent with the eutectic point of LA and SA, as shown in Figure 6. Therefore, a certain proportion of the EG-to-PCM ratio has little effect on its phase transition temperature, and the latent heat of phase transition can fluctuate. The melting point, solidification point, and phase transition enthalpy remained stable when the EG content was greater than 5 wt.%.

Because the DSC process is carried out under the condition of linear temperature change, the heat flow rate, *δQ*/d*T*, is directly proportional to the instantaneous specific heat capacity of the sample, *C*_p_. According to the DSC principle and the test data, the specific heat capacity of PCMs could be described and calculated by Equations (1) and (2).

(1) $$\frac{\delta Q}{\mathrm{dt}}=m\cdot C_{p}\cdot \frac{dT}{dt}$$

(2) $$C_{p}=\frac{1}{m}\cdot \left(\frac{\delta Q}{\mathrm{dt}}/\frac{dT}{dt}\right)=\frac{1}{m\cdot \beta }\cdot \frac{\delta Q}{\mathrm{dt}}$$

where *m* is the sample quality, *m* = 1 g; *β* is the temperature rate, *β* = d*T*/dt = 1 °C/min; and *δQ*/dt is the heat flux rate, w/g. 

**Figure 9:**
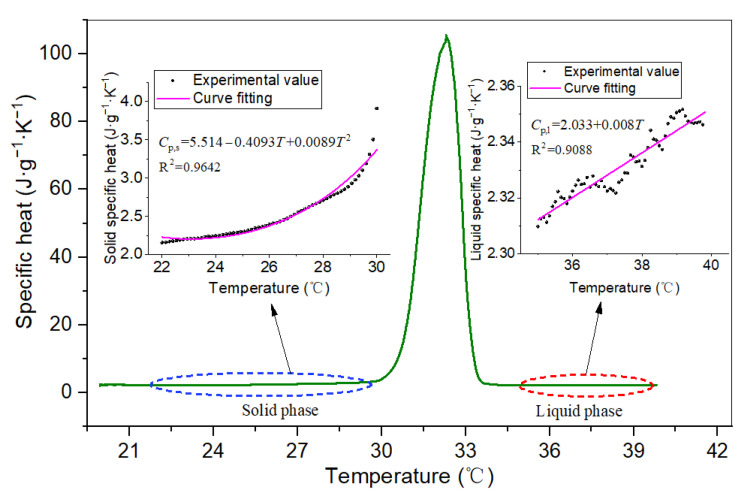
The specific heat capacity of PCM (0 wt.% EG).

Using the composite PCM with 0 wt.% EG as an example, Figure 9 shows the DSC curve of the specific capacity from the solid phase to the liquid phase during the endothermic process. In the solid- and liquid-phase stages, the specific heat capacity of the PCM slightly increased with temperature. The polynomial curve fitting between the specific heat and temperature was obtained, the average solid-state specific heat capacity was *C*_P,s_ = 2.371 J·g^−1^·k^−1^ and that of the liquid-state was *C*_P,l_ = 2.328 J·g^−1^·k^−1^.

**Figure 10:**
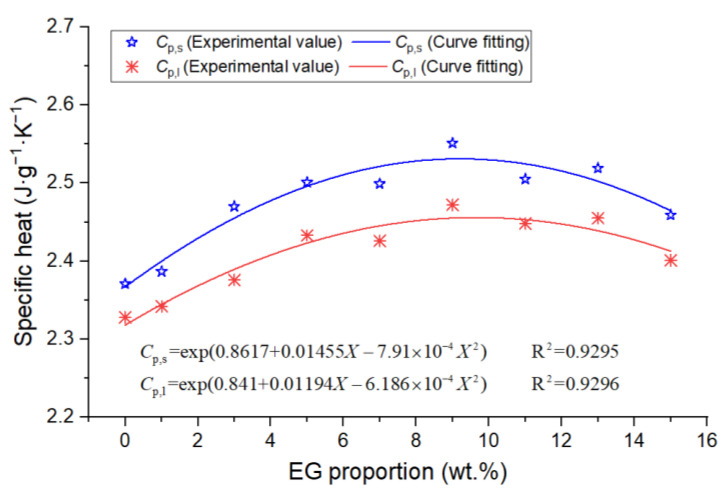
The specific heat capacity of PCMs.

Similarly, the average solid-state specific heat capacity and that of the liquid state of the other eight DSC samples were calculated, as shown in Figure 10. It can be seen that the specific heat capacity of the composite PCMs showed a trend of increasing first and then decreasing, reaching the maximum at around 9 wt.% EG.

### 3.3. TPS Results

**Figure 11:**
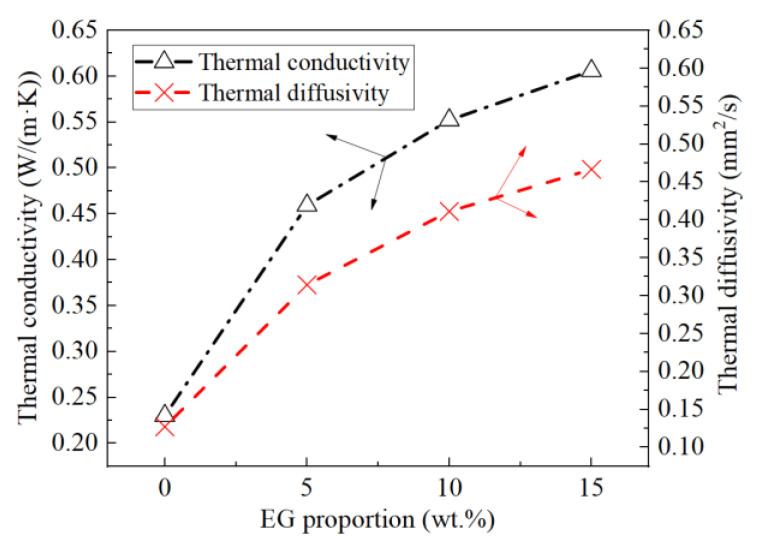
Thermal conductivity and thermal diffusivity.

Due to the porous structure properties of expanded graphite, PCMs can be adsorbed and filled in micropores. Moreover, because the thermal conductivity of EG (about 90 W·m^−1^·k^−1^) is much higher than that of the eutectic mixed acid, the thermal conductivity of composite PCMs is significantly improved. Figure 11 shows the TPS results of four test samples. The results showed that the thermal conductivity and diffusivity of the composite PCMs significantly increased with the EG proportion. In particular, when the EG proportion was 10 and 15 wt.%, the thermal conductivity of the composite PCMs was 2.4 and 2.6 times greater than that of the eutectic mixed acid, respectively, and the thermal diffusivity of the composite PCMs was 3.2 and 3.7 times greater than that of the eutectic mixed acid, respectively.

### 3.4. Low-Temperature Thermal Energy Storage Experiment Results

The development, preparation, and accurate measurement of the basic parameters of PCMs are significant. However, the heat storage and release performance in practical applications is even more critical. Therefore, measuring the heat storage and release effects of PCMs in real heat reservoirs is necessary.

The four heat reservoirs had the same test conditions. Moreover, as mentioned above, the composite PCM with 10 wt.% EG had the advantages in terms of phase transition enthalpy, specific heat capacity, and thermal conductivity. Using 0 wt.% EG PCMs as an example, we summarized and counted the four experimental results.

The set temperature of the thermostatic water bath was 40 °C under the heat storage condition and 16 °C under the heat release condition; there was a natural cooling stage in the middle, which was the operation of switching from 40 to 16 °C.

**Figure 12:**
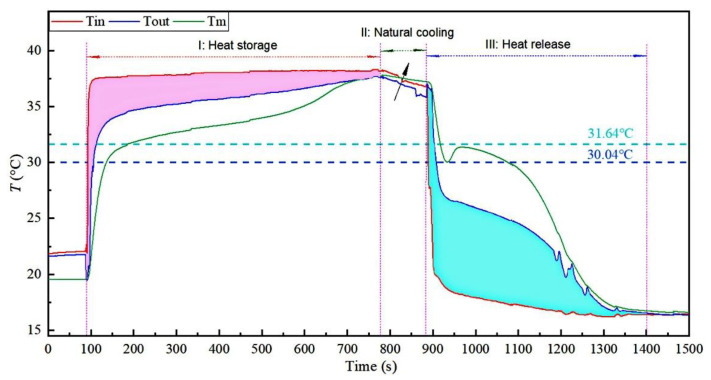
Temperature distribution of water and PCM (10 wt.% EG).

Figure 12 shows the temperature distribution of the inlet and outlet water (Tin: inlet water temperature, and Tout: outlet water temperature) and PCM (Tm: average temperature of 13 PCM sensors).

In the heat storage stage, the temperatures rapidly rose and entered the heat storage process with the system’s start-up. At 600 s, the PCM temperature showed an apparent rising trend, which indicated that the heat reservoir had completed the latent heat storage stage and entered the sensible heat storage stage. At 700 s, the temperature of the PCM was very close to the temperature of the water outlet, which indicated that the heat accumulator had completed the heat reservoir process. The hot-pink area represents the heat storage capacity, calculated from the inlet and outlet water temperature and flow.

In the process of switching the thermostatic water bath from 40 to 16 °C, the PCM’s temperature remained unchanged.

The exothermic process started at 880 s. Due to the significant temperature difference, the PCM temperature appeared at about 1.6 °C supercooling and then quickly entered the exothermic stage. At 1120 s, the PCM temperature showed an apparent downward trend, which indicated that the heat reservoir had completed the latent heat exothermic stage and entered the sensible heat exothermic stage. At 1350 s, the temperature of PCM was very close to the temperature of the water outlet, which indicated that the heat reservoir had completed the heat release process. The cyan area represents the heat release capacity, calculated from the inlet and outlet water temperature and flow.

In Figure 12, the heat storage and release times of the heat reservoir can also be separately observed in the hot-pink and cyan areas, respectively.

**Figure 13:**
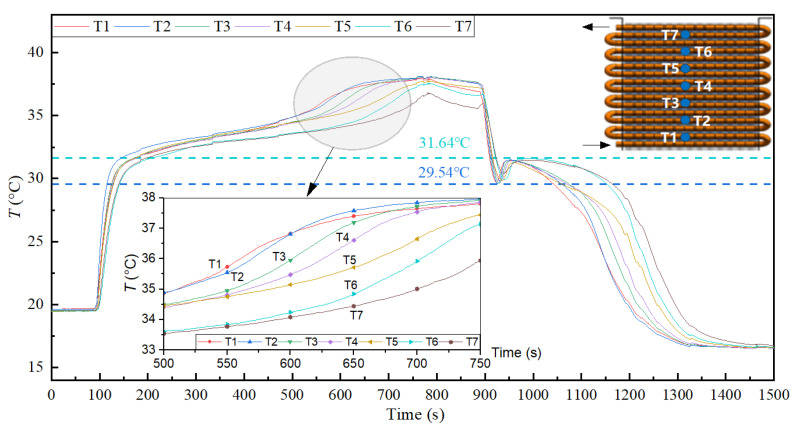
Longitudinal temperature distribution of PCM (10 wt.% EG).

**Figure 14:**
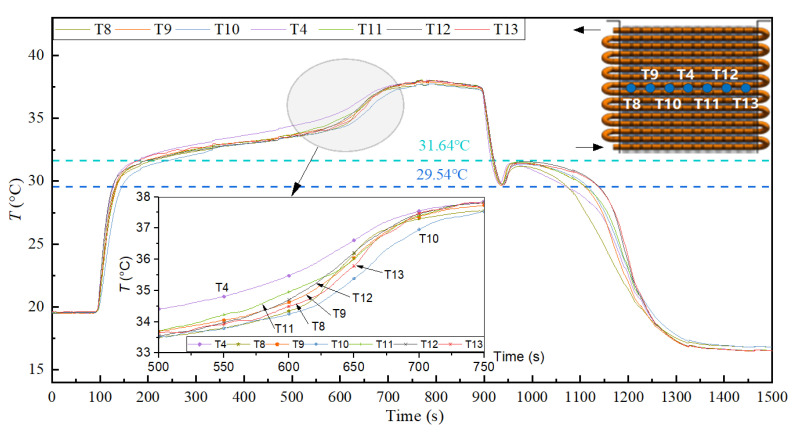
Transverse temperature distribution of PCM (10 wt.% EG).

Furthermore, the longitudinal temperature distribution of PCM was consistent with the direction of thermal fluid flow; the temperature successively decreased from T1 to T7, as shown in Figure 13. However, the transverse temperature distribution of PCM showed little difference in the flow direction of the same pipe side, as shown in Figure 14. The longitudinal and transverse data provided the calculation accuracy and basis for the performance of the heat reservoir.

**Figure 15:**
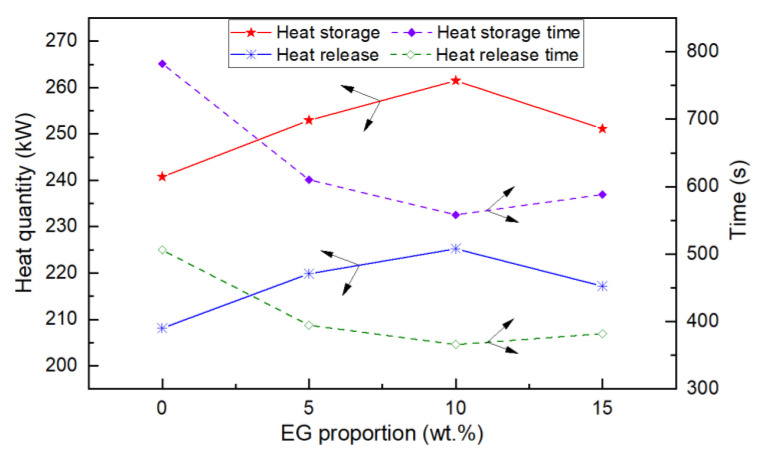
Heat storage, heat release, and time statistics.

As a result, through the statistics and calculation of the experimental data, the heat storage, heat storage time, heat release, and heat release time of the heat reservoir could be obtained. Similarly, the experimental data of the other three kinds of PCM were statistically calculated; the heat storage, heat release, and time of the heat reservoir are shown in Figure 15. It can be seen that, under the same conditions, when the ratio of expanded graphite was 10 wt.%, the heat storage capacity and heat release capacity of the heat reservoir were the largest, and the heat storage time and heat release time were the shortest.

In summary, because the finned-coil-type structure was used as the heat reservoir, the high thermal conductivity of brass tubes and aluminum fins and the dense arrangement of fins provided an excellent heat transfer interface for the rapid accumulation and release of low- and ultra-low-grade heat sources.

## 4. Conclusions

In this study, the eutectic point of lauric acid and stearic acid was tested by the step cooling curve method. Composite PCMs with different mass ratios of expanded graphite were prepared and tested, then the finned-coil-type heat reservoirs with four composite PCMs were prepared and tested. The drew the following conclusions:

1. According to the step cooling curve, the eutectic point of lauric acid and stearic acid was 31.2 °C when the mass proportion of LA was 70 wt.% and that of SA was 30 wt.%.
2. Based on the DSC and TPS results, the properties of composite PCMs remained stable when the EG content was greater than 5 wt.%, and the specific heat capacity reached the maximum when the EG ratio was about 9%. The thermal conductivity and thermal diffusion coefficient of the composite PCMs (10−15 wt.%) increased by 2.4–2.6 times and 3.2–3.7 times compared with those of pure eutectic acid, respectively. This indicated EG could enhance heat conduction.
3. The experimental results of the finned-coil-type heat reservoirs showed that the optimum ratio of EG was 10 wt.%. The heat storage time was reduced by 20.4%, 8.1%, and 6.2% compared with the other three EG ratios; meanwhile, the heat release time was decreased by 19.3%, 6.7%, and 5.3%.

Therefore, the composite eutectic fatty acids PCMs with the finned-coil-type heat reservoirs may have promising applications to enhance the energy efficiency of low- and ultra-low-grade thermal energy storage.

## Figures and Tables

**Table 1:** Parameters of the PCMs.

**Material**	**Melting Temperature** **(°C)**	**Specific Heat** **(J·g^−1^·K^−1^)**	**Thermal Conductivity** **(W·m^−1^·K^−1^)**	**Latent Heat** **(J·g^−1^** **)**	**Density** **(g·m^−3^** **)**
LA	44.0–46.0	1.60	0.147	184.4	870.0
SA	55.0–69.0	2.35	0.172	259.0	941.0

**Table 2:** Net weight of PCM in 4 heat reservoirs.

PCMs	0 wt.% EG	5 wt.% EG	10 wt.% EG	15 wt.% EG
Net weight (g)	1428.2	1437.3	1444.8	1431.7

## Data Availability

The data presented in this study are available on request from the corresponding author. The data are not publicly available because they also form part of an ongoing study.
